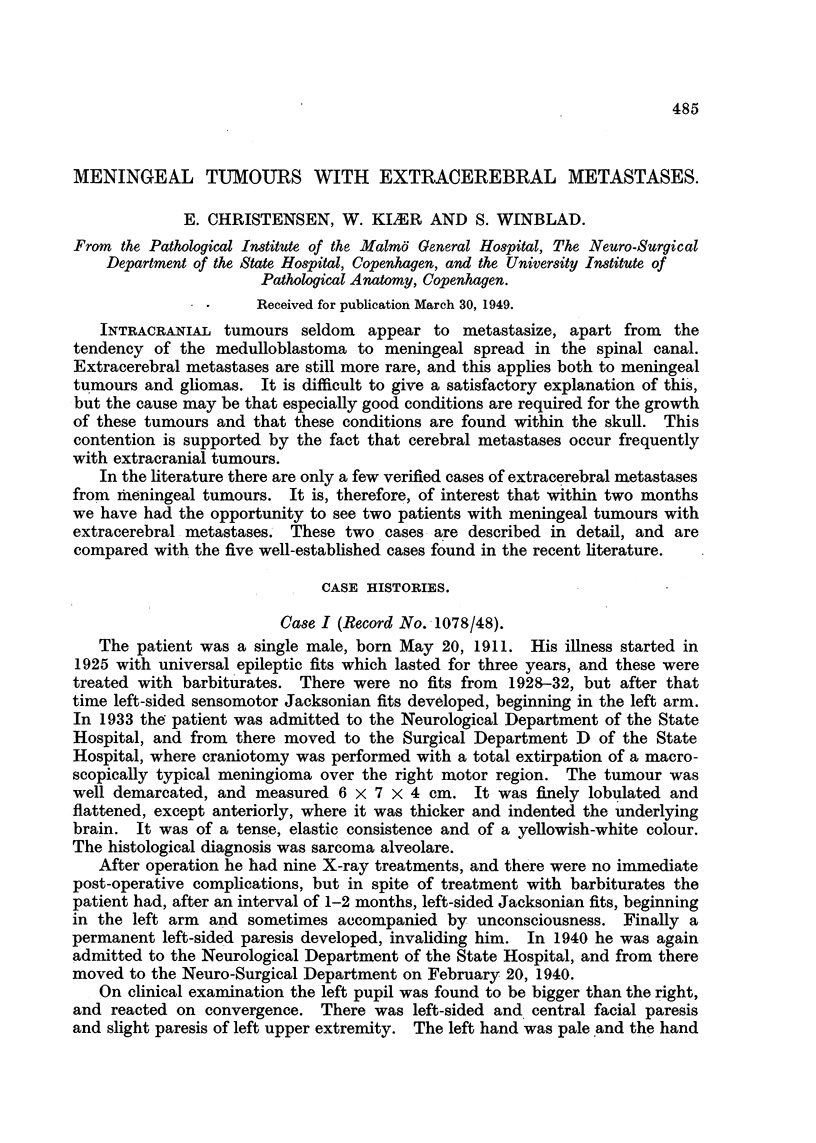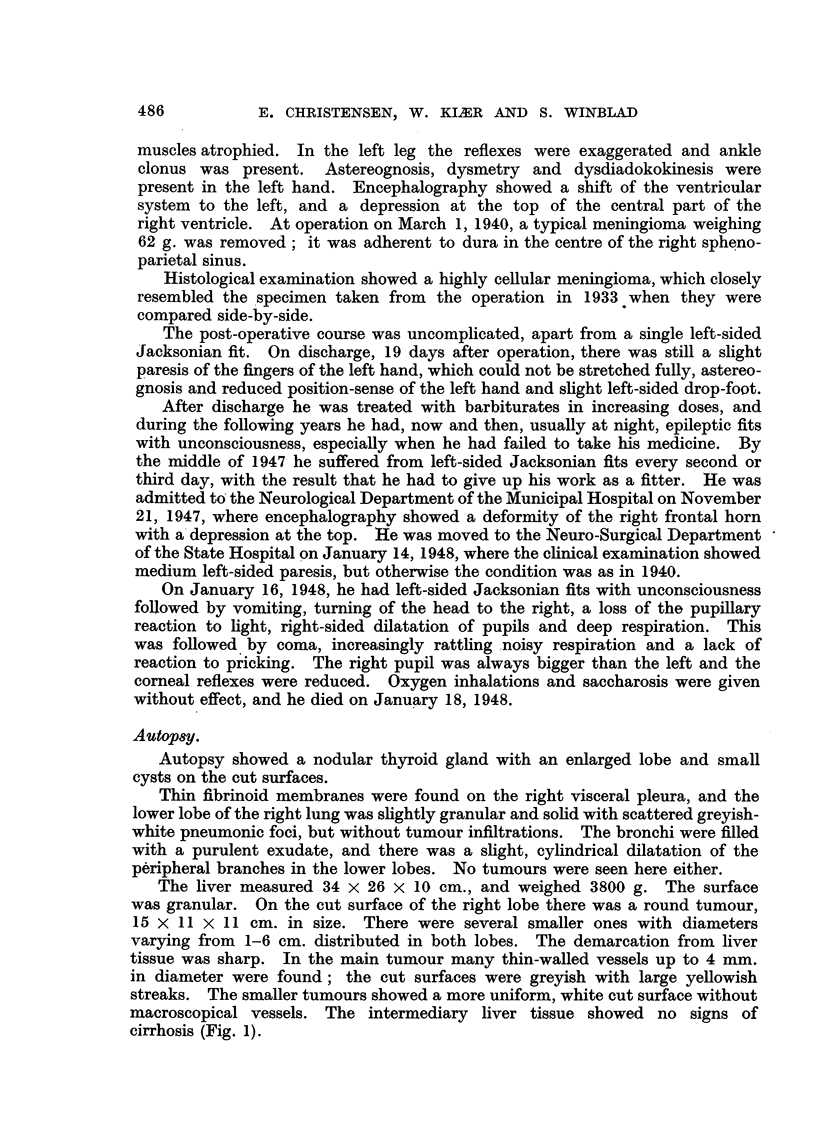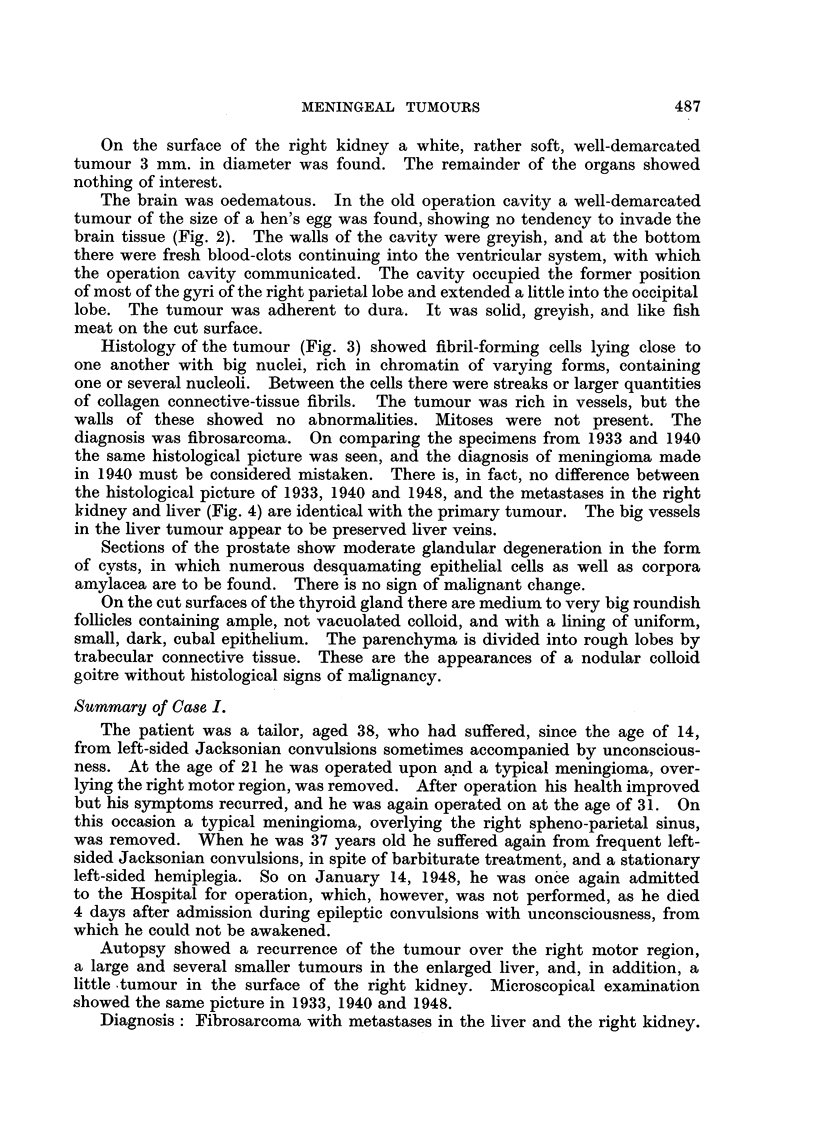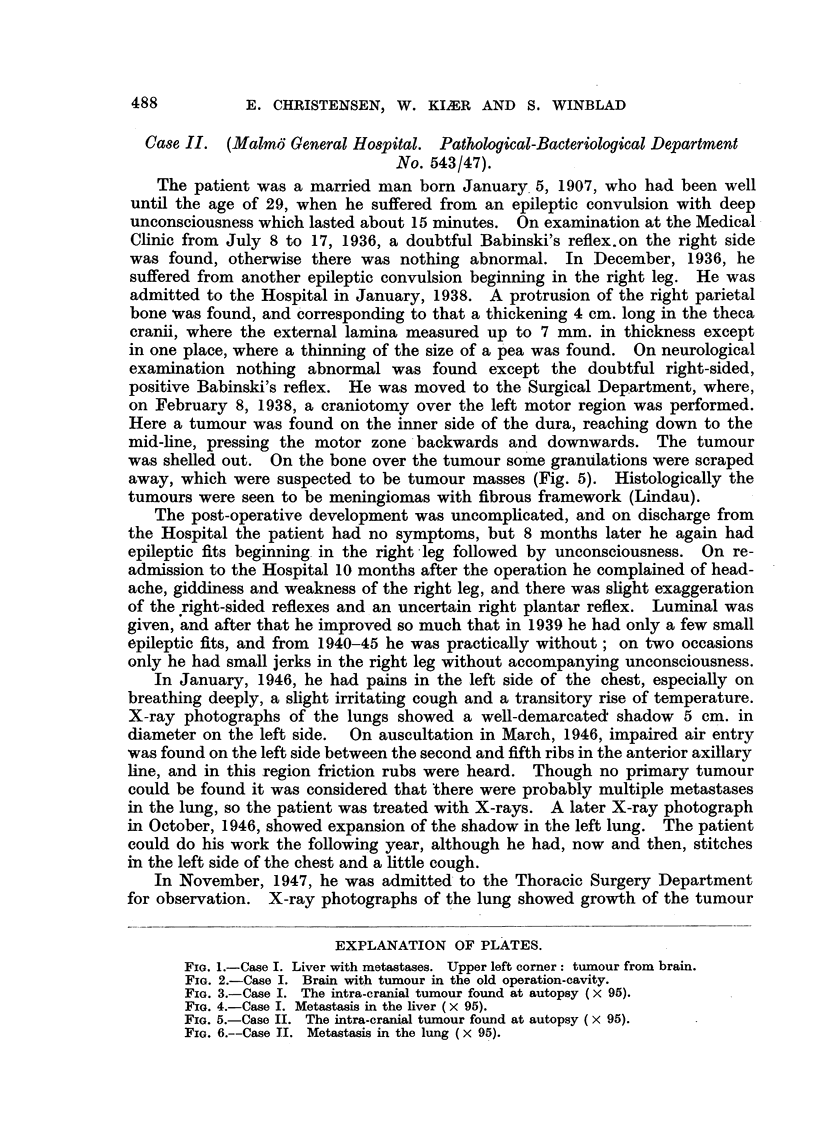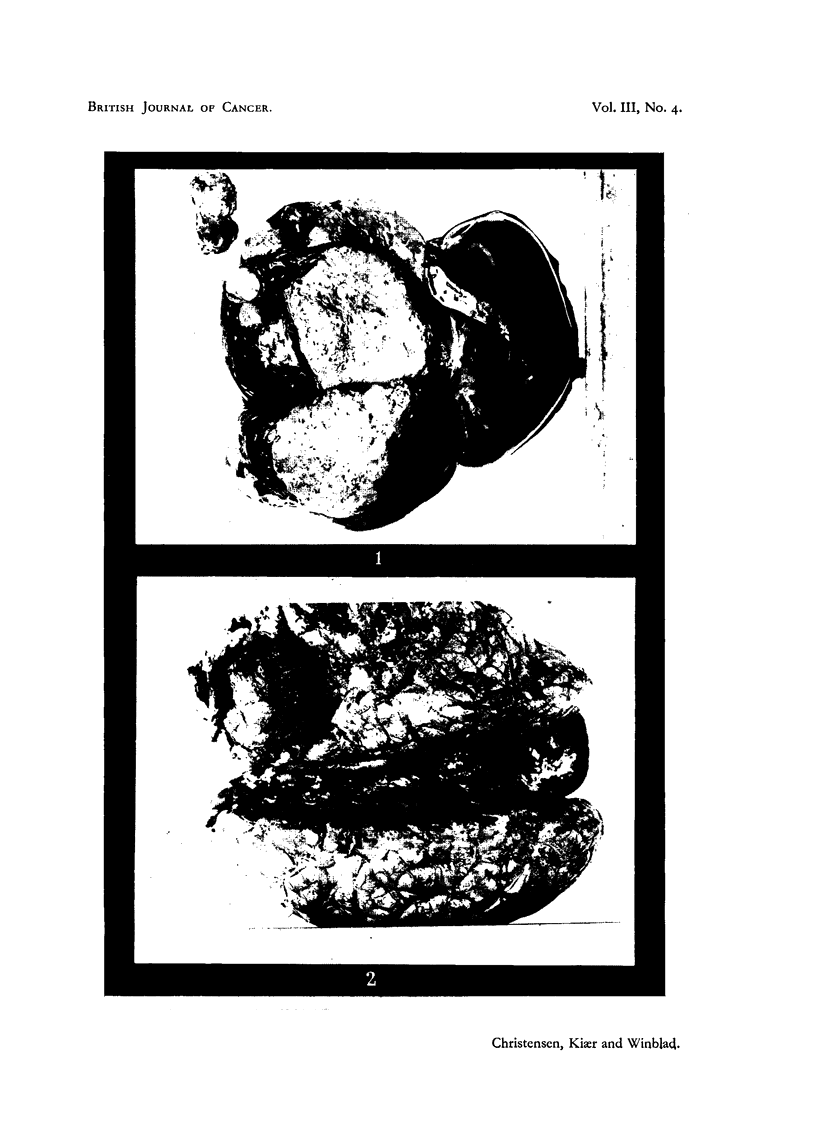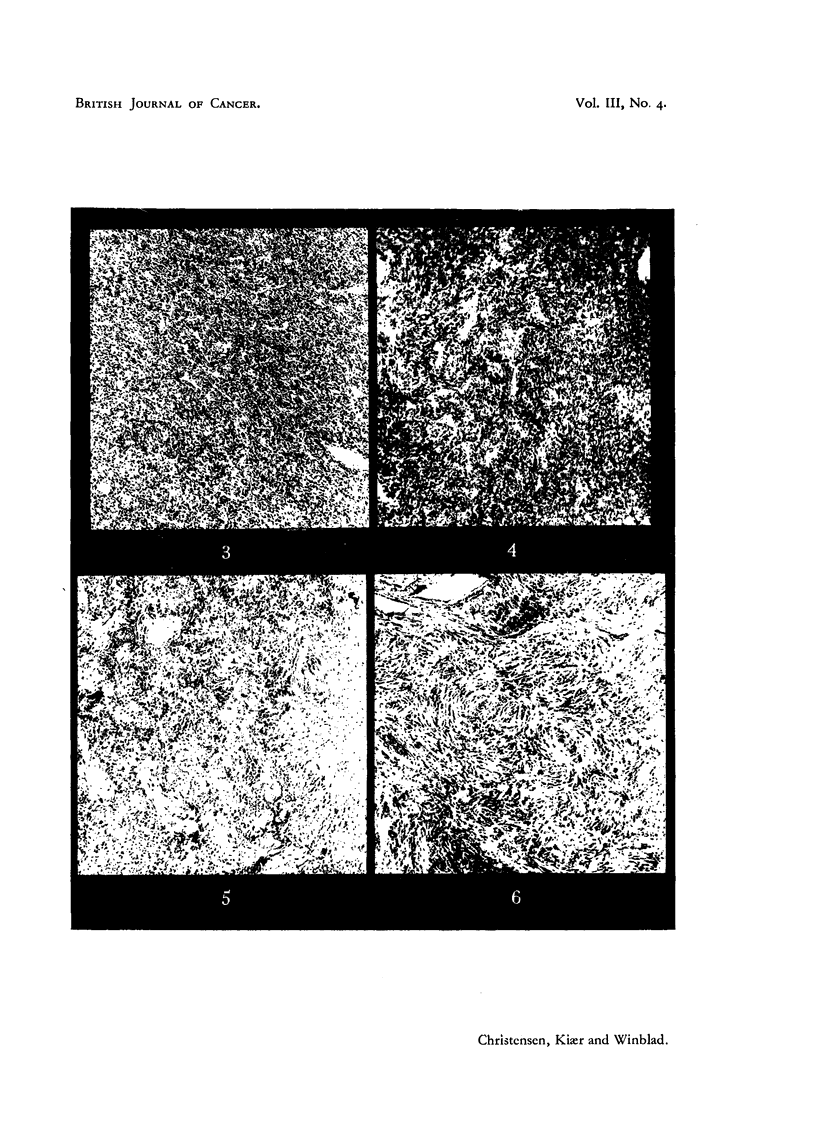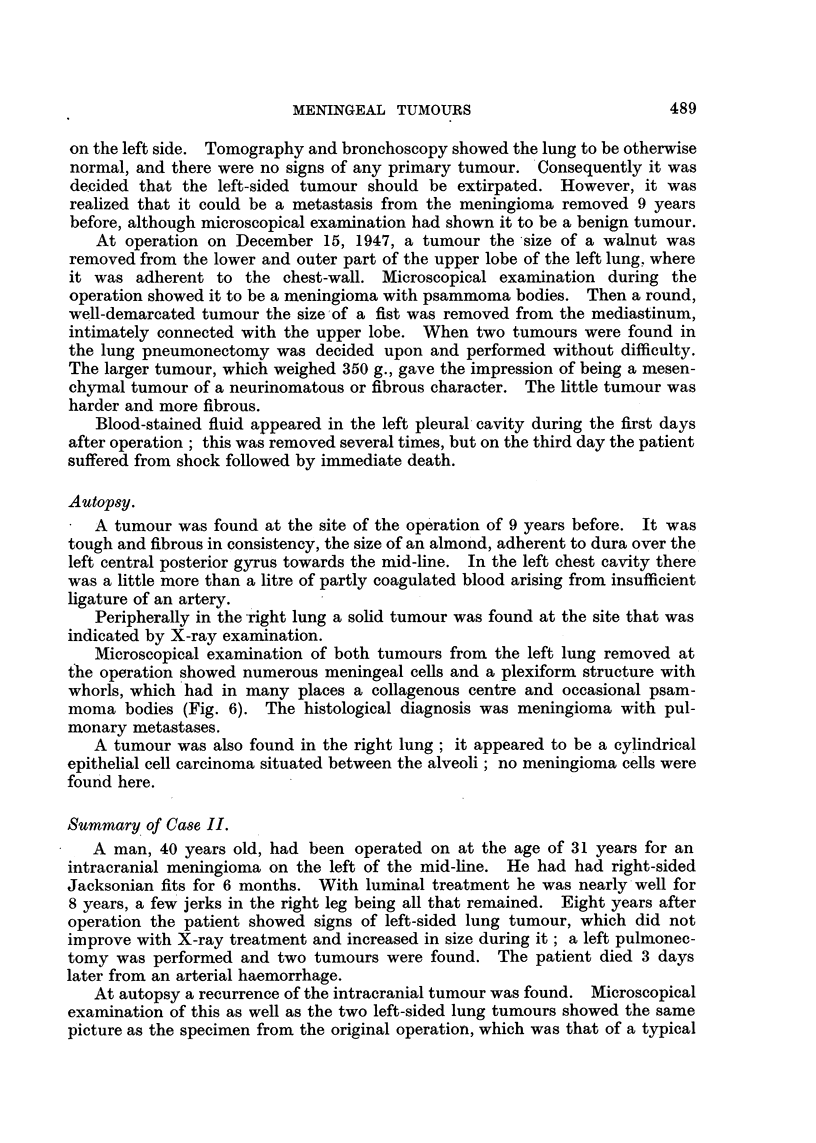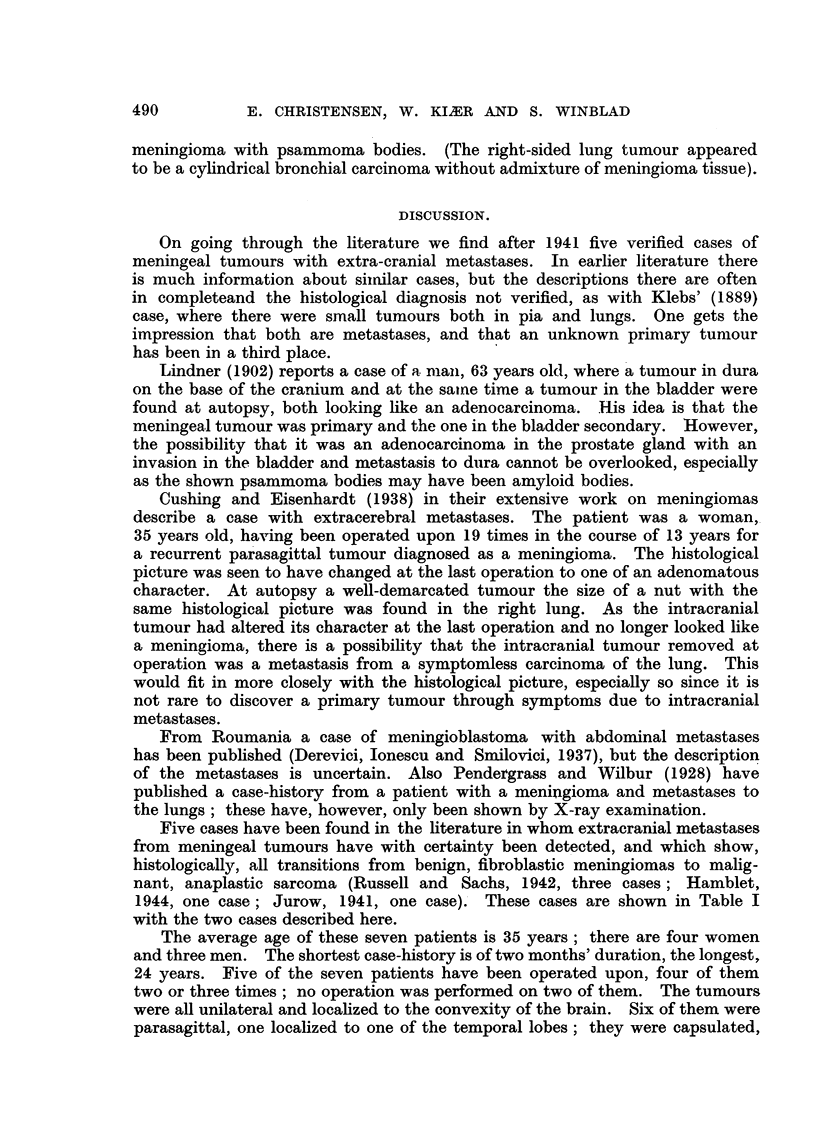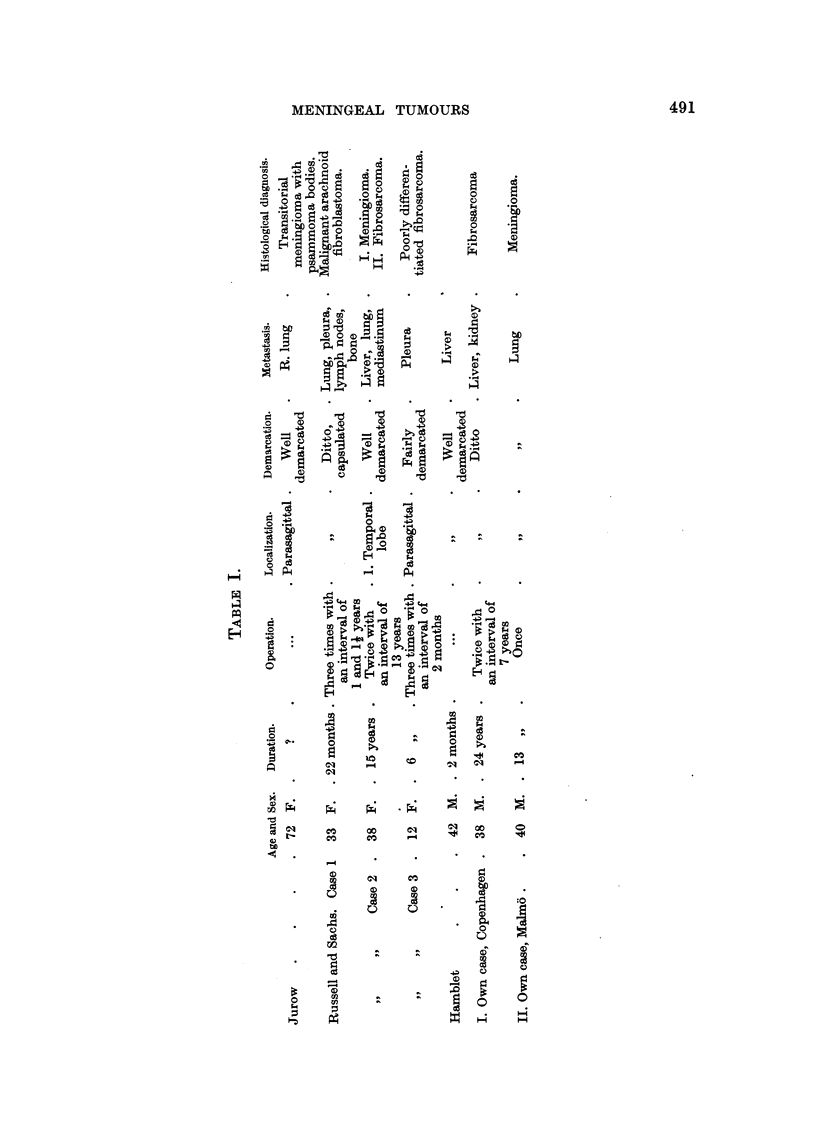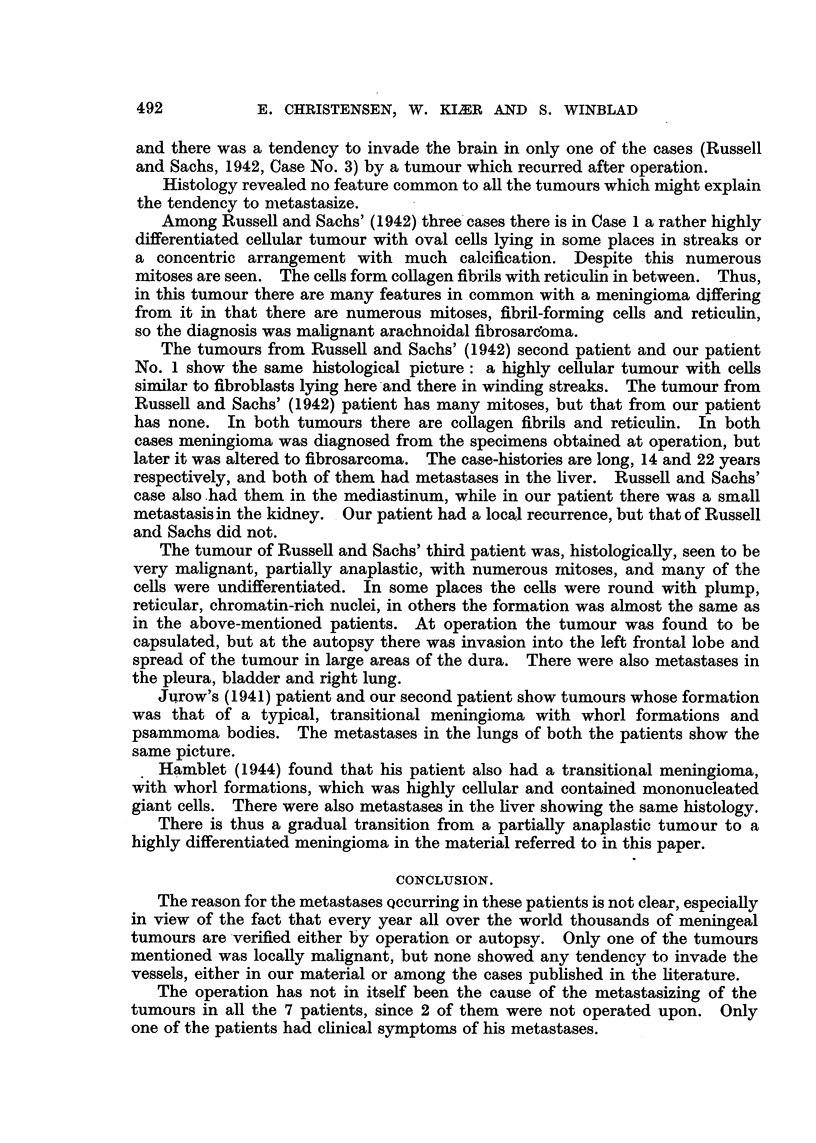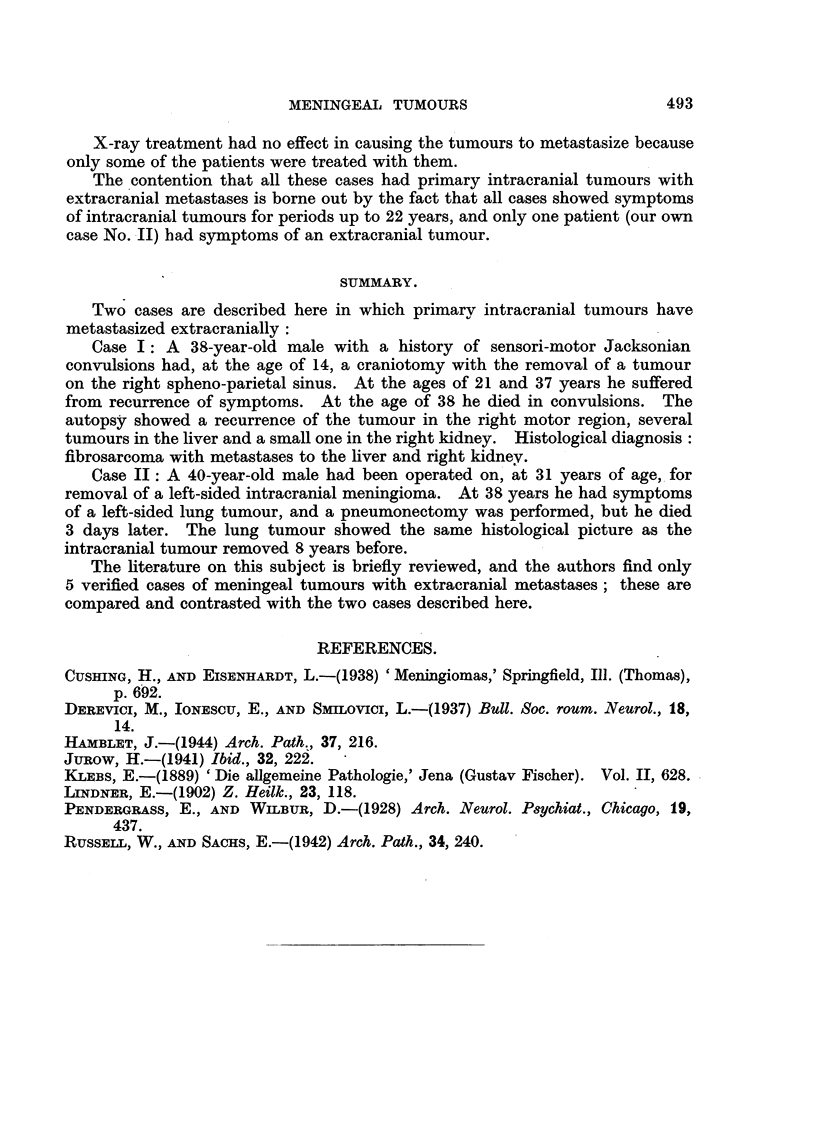# Meningeal Tumours with Extracerebral Metastases

**DOI:** 10.1038/bjc.1949.51

**Published:** 1949-12

**Authors:** E. Christensen, W. Kiær, S. Winblad

## Abstract

**Images:**


### MENINGEAL TUMOURS WITH EXTRACEREBRAL METASTASES.

E. CHRISTENSEN, W. KIÆR AND S. WINBLAD.

*From the Pathological Institute of the Malmö General Hospital, The Neuro-Surgical Department of the State Hospital, Copenhagen, and the University Institute of Pathological Anatomy, Copenhagen.*

Received for publication March 30, 1949.

INTRACRANIAL tumours seldom appear to metastasize, apart from the tendency of the medulloblastoma to meningeal spread in the spinal canal. Extracerebral metastases are still more rare, and this applies both to meningeal tumours and gliomas. It is difficult to give a satisfactory explanation of this, but the cause may be that especially good conditions are required for the growth of these tumours and that these conditions are found within the skull. This contention is supported by the fact that cerebral metastases occur frequently with extracranial tumours.

In the literature there are only a few verified cases of extracerebral metastases from meningeal tumours. It is, therefore, of interest that within two months we have had the opportunity to see two patients with meningeal tumours with extracerebral metastases. These two cases are described in detail, and are compared with the five well-established cases found in the recent literature.

### CASE HISTORIES.

### *Case I* (*Record No.* 1078/48).

The patient was a single male, born May 20, 1911. His illness started in 1925 with universal epileptic fits which lasted for three years, and these were treated with barbiturates. There were no fits from 1928–32, but after that time left-sided sensomotor Jacksonian fits developed, beginning in the left arm. In 1933 the patient was admitted to the Neurological Department of the State Hospital, and from there moved to the Surgical Department D of the State Hospital, where craniotomy was performed with a total extirpation of a macroscopically typical meningioma over the right motor region. The tumour was well demarcated, and measured 6 × 7 × 4 cm. It was finely lobulated and flattened, except anteriorly, where it was thicker and indented the underlying brain. It was of a tense, elastic consistence and of a yellowish-white colour. The histological diagnosis was sarcoma alveolare.

After operation he had nine X-ray treatments, and there were no immediate post-operative complications, but in spite of treatment with barbiturates the patient had, after an interval of 1–2 months, left-sided Jacksonian fits, beginning in the left arm and sometimes accompanied by unconsciousness. Finally a permanent left-sided paresis developed, invaliding him. In 1940 he was again admitted to the Neurological Department of the State Hospital, and from there moved to the Neuro-Surgical Department on February 20, 1940.

On clinical examination the left pupil was found to be bigger than the right, and reacted on convergence. There was left-sided and central facial paresis and slight paresis of left upper extremity. The left hand was pale and the hand muscles atrophied. In the left leg the reflexes were exaggerated and ankle clonus was present. Astereognosis, dysmetry and dysdiadokokinesis were present in the left hand. Encephalography showed a shift of the ventricular system to the left, and a depression at the top of the central part of the right ventricle. At operation on March 1, 1940, a typical meningioma weighing 62 g. was removed ; it was adherent to dura in the centre of the right spheno-parietal sinus.

Histological examination showed a highly cellular meningioma, which closely resembled the specimen taken from the operation in 1933 when they were compared side-by-side.

The post-operative course was uncomplicated, apart from a single left-sided Jacksonian fit. On discharge, 19 days after operation, there was still a slight paresis of the fingers of the left hand, which could not be stretched fully, astereognosis and reduced position-sense of the left hand and slight left-sided drop-foot.

After discharge he was treated with barbiturates in increasing doses, and during the following years he had, now and then, usually at night, epileptic fits with unconsciousness, especially when he had failed to take his medicine. By the middle of 1947 he suffered from left-sided Jacksonian fits every second or third day, with the result that he had to give up his work as a fitter. He was admitted to the Neurological Department of the Municipal Hospital on November 21, 1947, where encephalography showed a deformity of the right frontal horn with a depression at the top. He was moved to the Neuro-Surgical Department of the State Hospital on January 14, 1948, where the clinical examination showed medium left-sided paresis, but otherwise the condition was as in 1940.

On January 16, 1948, he had left-sided Jacksonian fits with unconsciousness followed by vomiting, turning of the head to the right, a loss of the pupillary reaction to light, right-sided dilatation of pupils and deep respiration. This was followed by coma, increasingly rattling noisy respiration and a lack of reaction to pricking. The right pupil was always bigger than the left and the corneal reflexes were reduced. Oxygen inhalations and saccharosis were given without effect, and he died on January 18, 1948.

*Autopsy.*

Autopsy showed a nodular thyroid gland with an enlarged lobe and small cysts on the cut surfaces.

Thin fibrinoid membranes were found on the right visceral pleura, and the lower lobe of the right lung was slightly granular and solid with scattered greyish-white pneumonic foci, but without tumour infiltrations. The bronchi were filled with a purulent exudate, and there was a slight, cylindrical dilatation of the peripheral branches in the lower lobes. No tumours were seen here either.

The liver measured 34 × 26 × 10 cm., and weighed 3800 g. The surface was granular. On the cut surface of the right lobe there was a round tumour, 15 × 11 × 11 cm. in size. There were several smaller ones with diameters varying from 1–6 cm. distributed in both lobes. The demarcation from liver tissue was sharp. In the main tumour many thin-walled vessels up to 4 mm. in diameter were found ; the cut surfaces were greyish with large yellowish streaks. The smaller tumours showed a more uniform, white cut surface without macroscopical vessels. The intermediary liver tissue showed no signs of cirrhosis (Fig. 1).

On the surface of the right kidney a white, rather soft, well-demarcated tumour 3 mm. in diameter was found. The remainder of the organs showed nothing of interest.

The brain was oedematous. In the old operation cavity a well-demarcated tumour of the size of a hen's egg was found, showing no tendency to invade the brain tissue (Fig. 2). The walls of the cavity were greyish, and at the bottom there were fresh blood-clots continuing into the ventricular system, with which the operation cavity communicated. The cavity occupied the former position of most of the gyri of the right parietal lobe and extended a little into the occipital lobe. The tumour was adherent to dura. It was solid, greyish, and like fish meat on the cut surface.

Histology of the tumour (Fig. 3) showed fibril-forming cells lying close to one another with big nuclei, rich in chromatin of varying forms, containing one or several nucleoli. Between the cells there were streaks or larger quantities of collagen connective-tissue fibrils. The tumour was rich in vessels, but the walls of these showed no abnormalities. Mitoses were not present. The diagnosis was fibrosarcoma. On comparing the specimens from 1933 and 1940 the same histological picture was seen, and the diagnosis of meningioma made in 1940 must be considered mistaken. There is, in fact, no difference between the histological picture of 1933, 1940 and 1948, and the metastases in the right kidney and liver (Fig. 4) are identical with the primary tumour. The big vessels in the liver tumour appear to be preserved liver veins.

Sections of the prostate show moderate glandular degeneration in the form of cysts, in which numerous desquamating epithelial cells as well as corpora amylacea are to be found. There is no sign of malignant change.

On the cut surfaces of the thyroid gland there are medium to very big roundish follicles containing ample, not vacuolated colloid, and with a lining of uniform, small, dark, cubal epithelium. The parenchyma is divided into rough lobes by trabecular connective tissue. These are the appearances of a nodular colloid goitre without histological signs of malignancy.

*Summary of Case I.*

The patient was a tailor, aged 38, who had suffered, since the age of 14, from left-sided Jacksonian convulsions sometimes accompanied by unconsciousness. At the age of 21 he was operated upon and a typical meningioma, overlying the right motor region, was removed. After operation his health improved but his symptoms recurred, and he was again operated on at the age of 31. On this occasion a typical meningioma, overlying the right spheno-parietal sinus, was removed. When he was 37 years old he suffered again from frequent left-sided Jacksonian convulsions, in spite of barbiturate treatment, and a stationary left-sided hemiplegia. So on January 14, 1948, he was once again admitted to the Hospital for operation, which, however, was not performed, as he died 4 days after admission during epileptic convulsions with unconsciousness, from which he could not be awakened.

Autopsy showed a recurrence of the tumour over the right motor region, a large and several smaller tumours in the enlarged liver, and, in addition, a little tumour in the surface of the right kidney. Microscopical examination showed the same picture in 1933, 1940 and 1948.

Diagnosis : Fibrosarcoma with metastases in the liver and the right kidney.

*Case II.* (*Malmö General Hospital. Pathological-Bacteriological Department No.* 543/47).

The patient was a married man born January 5, 1907, who had been well until the age of 29, when he suffered from an epileptic convulsion with deep unconsciousness which lasted about 15 minutes. On examination at the Medical Clinic from July 8 to 17, 1936, a doubtful Babinski's reflex on the right side was found, otherwise there was nothing abnormal. In December, 1936, he suffered from another epileptic convulsion beginning in the right leg. He was admitted to the Hospital in January, 1938. A protrusion of the right parietal bone was found, and corresponding to that a thickening 4 cm. long in the theca cranii, where the external lamina measured up to 7 mm. in thickness except in one place, where a thinning of the size of a pea was found. On neurological examination nothing abnormal was found except the doubtful right-sided, positive Babinski's reflex. He was moved to the Surgical Department, where, on February 8, 1938, a craniotomy over the left motor region was performed. Here a tumour was found on the inner side of the dura, reaching down to the mid-line, pressing the motor zone backwards and downwards. The tumour was shelled out. On the bone over the tumour some granulations were scraped away, which were suspected to be tumour masses (Fig. 5). Histologically the tumours were seen to be meningiomas with fibrous framework (Lindau).

The post-operative development was uncomplicated, and on discharge from the Hospital the patient had no symptoms, but 8 months later he again had epileptic fits beginning in the right leg followed by unconsciousness. On re-admission to the Hospital 10 months after the operation he complained of head-ache, giddiness and weakness of the right leg, and there was slight exaggeration of the right-sided reflexes and an uncertain right plantar reflex. Luminal was given, and after that he improved so much that in 1939 he had only a few small epileptic fits, and from 1940–45 he was practically without; on two occasions only he had small jerks in the right leg without accompanying unconsciousness.

In January, 1946, he had pains in the left side of the chest, especially on breathing deeply, a slight irritating cough and a transitory rise of temperature. X-ray photographs of the lungs showed a well-demarcated shadow 5 cm. in diameter on the left side. On auscultation in March, 1946, impaired air entry was found on the left side between the second and fifth ribs in the anterior axillary line, and in this region friction rubs were heard. Though no primary tumour could be found it was considered that there were probably multiple metastases in the lung, so the patient was treated with X-rays. A later X-ray photograph in October, 1946, showed expansion of the shadow in the left lung. The patient could do his work the following year, although he had, now and then, stitches in the left side of the chest and a little cough.

In November, 1947, he was admitted to the Thoracic Surgery Department for observation. X-ray photographs of the lung showed growth of the tumour

---

EXPLANATION OF PLATES.

FIG. 1.—Case I. Liver with metastases. Upper left corner: tumour from brain.
FIG. 2.—Case I. Brain with tumour in the old operation-cavity.
FIG. 3.—Case I. The intra-cranial tumour found at autopsy (× 95).
FIG. 4.—Case I. Metastasis in the liver (× 95).
FIG. 5.—Case II. The intra-cranial tumour found at autopsy (× 95).
FIG. 6.—Case II. Metastasis in the lung (× 95).

1

2

Christensen, Kiær and Winblad.

Christensen, Kiær and Winblad.

on the left side. Tomography and bronchoscopy showed the lung to be otherwise normal, and there were no signs of any primary tumour. Consequently it was decided that the left-sided tumour should be extirpated. However, it was realized that it could be a metastasis from the meningioma removed 9 years before, although microscopical examination had shown it to be a benign tumour.

At operation on December 15, 1947, a tumour the size of a walnut was removed from the lower and outer part of the upper lobe of the left lung, where it was adherent to the chest-wall. Microscopical examination during the operation showed it to be a meningioma with psammoma bodies. Then a round, well-demarcated tumour the size of a fist was removed from the mediastinum, intimately connected with the upper lobe. When two tumours were found in the lung pneumonectomy was decided upon and performed without difficulty. The larger tumour, which weighed 350 g., gave the impression of being a mesenchymal tumour of a neurinomatous or fibrous character. The little tumour was harder and more fibrous.

Blood-stained fluid appeared in the left pleural cavity during the first days after operation ; this was removed several times, but on the third day the patient suffered from shock followed by immediate death.

*Autopsy.*

A tumour was found at the site of the operation of 9 years before. It was tough and fibrous in consistency, the size of an almond, adherent to dura over the left central posterior gyrus towards the mid-line. In the left chest cavity there was a little more than a litre of partly coagulated blood arising from insufficient ligature of an artery.

Peripherally in the right lung a solid tumour was found at the site that was indicated by X-ray examination.

Microscopical examination of both tumours from the left lung removed at the operation showed numerous meningeal cells and a plexiform structure with whorls, which had in many places a collagenous centre and occasional psammoma bodies (Fig. 6). The histological diagnosis was meningioma with pulmonary metastases.

A tumour was also found in the right lung ; it appeared to be a cylindrical epithelial cell carcinoma situated between the alveoli ; no meningioma cells were found here.

*Summary of Case II.*

A man, 40 years old, had been operated on at the age of 31 years for an intracranial meningioma on the left of the mid-line. He had had right-sided Jacksonian fits for 6 months. With luminal treatment he was nearly well for 8 years, a few jerks in the right leg being all that remained. Eight years after operation the patient showed signs of left-sided lung tumour, which did not improve with X-ray treatment and increased in size during it ; a left pulmonectomy was performed and two tumours were found. The patient died 3 days later from an arterial haemorrhage.

At autopsy a recurrence of the intracranial tumour was found. Microscopical examination of this as well as the two left-sided lung tumours showed the same picture as the specimen from the original operation, which was that of a typical meningioma with psammoma bodies. (The right-sided lung tumour appeared to be a cylindrical bronchial carcinoma without admixture of meningioma tissue).

### DISCUSSION.

On going through the literature we find after 1941 five verified cases of meningeal tumours with extra-cranial metastases. In earlier literature there is much information about similar cases, but the descriptions there are often in completeand the histological diagnosis not verified, as with Klebs' (1889) case, where there were small tumours both in pia and lungs. One gets the impression that both are metastases, and that an unknown primary tumour has been in a third place.

Lindner (1902) reports a case of a man, 63 years old, where a tumour in dura on the base of the cranium and at the same time a tumour in the bladder were found at autopsy, both looking like an adenocarcinoma. His idea is that the meningeal tumour was primary and the one in the bladder secondary. However, the possibility that it was an adenocarcinoma in the prostate gland with an invasion in the bladder and metastasis to dura cannot be overlooked, especially as the shown psammoma bodies may have been amyloid bodies.

Cushing and Eisenhardt (1938) in their extensive work on meningiomas describe a case with extracerebral metastases. The patient was a woman, 35 years old, having been operated upon 19 times in the course of 13 years for a recurrent parasagittal tumour diagnosed as a meningioma. The histological picture was seen to have changed at the last operation to one of an adenomatous character. At autopsy a well-demarcated tumour the size of a nut with the same histological picture was found in the right lung. As the intracranial tumour had altered its character at the last operation and no longer looked like a meningioma, there is a possibility that the intracranial tumour removed at operation was a metastasis from a symptomless carcinoma of the lung. This would fit in more closely with the histological picture, especially so since it is not rare to discover a primary tumour through symptoms due to intracranial metastases.

From Roumania a case of meningioblastoma with abdominal metastases has been published (Derevici, Ionescu and Smilovici, 1937), but the description of the metastases is uncertain. Also Pendergrass and Wilbur (1928) have published a case-history from a patient with a meningioma and metastases to the lungs ; these have, however, only been shown by X-ray examination.

Five cases have been found in the literature in whom extracranial metastases from meningeal tumours have with certainty been detected, and which show, histologically, all transitions from benign, fibroblastic meningiomas to malignant, anaplastic sarcoma (Russell and Sachs, 1942, three cases ; Hamblet, 1944, one case ; Jurow, 1941, one case). These cases are shown in Table I with the two cases described here.

The average age of these seven patients is 35 years ; there are four women and three men. The shortest case-history is of two months' duration, the longest, 24 years. Five of the seven patients have been operated upon, four of them two or three times ; no operation was performed on two of them. The tumours were all unilateral and localized to the convexity of the brain. Six of them were parasagittal, one localized to one of the temporal lobes ; they were capsulated,

TABLE I.

| | Age and Sex. | Duration. | Operation. | Localization. | Demarcation. | Metastasis. | Histological diagnosis. |
|---|---|---|---|---|---|---|---|
| Jurow | 72 F. | ? | ... | Parasagittal | Well demarcated | R. lung | Transitorial meningioma with psammoma bodies. |
| Russell and Sachs. Case 1 | 33 F. | 22 months | Three times with an interval of 1 and 1½ years | ,, | Ditto, capsulated | Lung, pleura, lymph nodes, bone | Malignant arachnoid fibroblastoma. |
| ,, ,, Case 2 | 38 F. | 15 years | Twice with an interval of 13 years | 1. Temporal lobe | Well demarcated | Liver, lung, mediastinum | I. Meningioma. II. Fibrosarcoma. |
| ,, ,, Case 3 | 12 F. | 6 ,, | Three times with an interval of 2 months | Parasagittal | Fairly demarcated | Pleura | Poorly differentiated fibrosarcoma. |
| Hamblet | 42 M. | 2 months | ... | ,, | Well demarcated | Liver | |
| I. Own case, Copenhagen | 38 M. | 24 years | Twice with an interval of 7 years | ,, | Ditto | Liver, kidney | Fibrosarcoma |
| II. Own case, Malmö | 40 M. | 13 ,, | Once | ,, | ,, | Lung | Meningioma. |

and there was a tendency to invade the brain in only one of the cases (Russell and Sachs, 1942, Case No. 3) by a tumour which recurred after operation.

Histology revealed no feature common to all the tumours which might explain the tendency to metastasize.

Among Russell and Sachs' (1942) three cases there is in Case 1 a rather highly differentiated cellular tumour with oval cells lying in some places in streaks or a concentric arrangement with much calcification. Despite this numerous mitoses are seen. The cells form collagen fibrils with reticulin in between. Thus, in this tumour there are many features in common with a meningioma differing from it in that there are numerous mitoses, fibril-forming cells and reticulin, so the diagnosis was malignant arachnoidal fibrosarcoma.

The tumours from Russell and Sachs' (1942) second patient and our patient No. 1 show the same histological picture : a highly cellular tumour with cells similar to fibroblasts lying here and there in winding streaks. The tumour from Russell and Sachs' (1942) patient has many mitoses, but that from our patient has none. In both tumours there are collagen fibrils and reticulin. In both cases meningioma was diagnosed from the specimens obtained at operation, but later it was altered to fibrosarcoma. The case-histories are long, 14 and 22 years respectively, and both of them had metastases in the liver. Russell and Sachs' case also had them in the mediastinum, while in our patient there was a small metastasis in the kidney. Our patient had a local recurrence, but that of Russell and Sachs did not.

The tumour of Russell and Sachs' third patient was, histologically, seen to be very malignant, partially anaplastic, with numerous mitoses, and many of the cells were undifferentiated. In some places the cells were round with plump, reticular, chromatin-rich nuclei, in others the formation was almost the same as in the above-mentioned patients. At operation the tumour was found to be capsulated, but at the autopsy there was invasion into the left frontal lobe and spread of the tumour in large areas of the dura. There were also metastases in the pleura, bladder and right lung.

Jurow's (1941) patient and our second patient show tumours whose formation was that of a typical, transitional meningioma with whorl formations and psammoma bodies. The metastases in the lungs of both the patients show the same picture.

Hamblet (1944) found that his patient also had a transitional meningioma, with whorl formations, which was highly cellular and contained mononucleated giant cells. There were also metastases in the liver showing the same histology.

There is thus a gradual transition from a partially anaplastic tumour to a highly differentiated meningioma in the material referred to in this paper.

### CONCLUSION.

The reason for the metastases occurring in these patients is not clear, especially in view of the fact that every year all over the world thousands of meningeal tumours are verified either by operation or autopsy. Only one of the tumours mentioned was locally malignant, but none showed any tendency to invade the vessels, either in our material or among the cases published in the literature.

The operation has not in itself been the cause of the metastasizing of the tumours in all the 7 patients, since 2 of them were not operated upon. Only one of the patients had clinical symptoms of his metastases.

X-ray treatment had no effect in causing the tumours to metastasize because only some of the patients were treated with them.

The contention that all these cases had primary intracranial tumours with extracranial metastases is borne out by the fact that all cases showed symptoms of intracranial tumours for periods up to 22 years, and only one patient (our own case No. II) had symptoms of an extracranial tumour.

### SUMMARY.

Two cases are described here in which primary intracranial tumours have metastasized extracranially :

Case I : A 38-year-old male with a history of sensori-motor Jacksonian convulsions had, at the age of 14, a craniotomy with the removal of a tumour on the right spheno-parietal sinus. At the ages of 21 and 37 years he suffered from recurrence of symptoms. At the age of 38 he died in convulsions. The autopsy showed a recurrence of the tumour in the right motor region, several tumours in the liver and a small one in the right kidney. Histological diagnosis : fibrosarcoma with metastases to the liver and right kidney.

Case II : A 40-year-old male had been operated on, at 31 years of age, for removal of a left-sided intracranial meningioma. At 38 years he had symptoms of a left-sided lung tumour, and a pneumonectomy was performed, but he died 3 days later. The lung tumour showed the same histological picture as the intracranial tumour removed 8 years before.

The literature on this subject is briefly reviewed, and the authors find only 5 verified cases of meningeal tumours with extracranial metastases ; these are compared and contrasted with the two cases described here.

### REFERENCES.

Cushing, H., and Eisenhardt, L.—(1938) 'Meningiomas,' Springfield, Ill. (Thomas), p. 692.

Derevici, M., Ionescu, E., and Smilovici, L.—(1937) *Bull. Soc. roum. Neurol.*, **18**, 14.

Hamblet, J.—(1944) *Arch. Path.*, **37**, 216.

Jurow, H.—(1941) *Ibid.*, **32**, 222.

Klebs, E.—(1889) 'Die allgemeine Pathologie,' Jena (Gustav Fischer). Vol. II, 628.

Lindner, E.—(1902) *Z. Heilk.*, **23**, 118.

Pendergrass, E., and Wilbur, D.—(1928) *Arch. Neurol. Psychiat., Chicago*, **19**, 437.

Russell, W., and Sachs, E.—(1942) *Arch. Path.*, **34**, 240.